# Brain FNDC5/Irisin Expression in Patients and Mouse Models of Major Depression

**DOI:** 10.1523/ENEURO.0256-22.2023

**Published:** 2023-02-13

**Authors:** Ricardo Lima-Filho, Juliana S. Fortuna, Danielle Cozachenco, Alinny R. Isaac, Natalia Lyra e Silva, Alice Saldanha, Luis E. Santos, Sergio T. Ferreira, Mychael V. Lourenco, Fernanda G. De Felice

**Affiliations:** 1 Institute of Medical Biochemistry Leopoldo de Meis, Federal University of Rio de Janeiro, 21941-902, Rio de Janeiro RJ, Brazil; 2 Institute of Biomedical Sciences, Federal University of Rio de Janeiro, 21941-902, Rio de Janeiro RJ, Brazil; 3 Institute of Biophysics Carlos Chagas Filho, Federal University of Rio de Janeiro, 21941-902, Rio de Janeiro RJ, Brazil; 4 Centre for Neurosciences Studies, Departments of Biomedical and Molecular Sciences, and Psychiatry, Queen’s University, Kingston, Ontario K7L 3N6, Canada; 5 D’Or Institute for Research and Education, Rio de Janeiro RJ, 22281-100, Brazil

**Keywords:** FNDC5, irisin, major depression, mood, mouse models

## Abstract

Major depressive disorder (MDD) is a major cause of disability in adults. MDD is both a comorbidity and a risk factor for Alzheimer’s disease (AD), and regular physical exercise has been associated with reduced incidence and severity of MDD and AD. Irisin is an exercise-induced myokine derived from proteolytic processing of fibronectin type III domain-containing protein 5 (FNDC5). FNDC5/irisin is reduced in the brains of AD patients and mouse models. However, whether brain FNDC5/irisin expression is altered in depression remains elusive. Here, we investigate changes in *fndc5* expression in postmortem brain tissue from MDD individuals and mouse models of depression. We found decreased *fndc5* expression in the MDD prefrontal cortex, both with and without psychotic traits. We further demonstrate that the induction of depressive-like behavior in male mice by lipopolysaccharide decreased *fndc5* expression in the frontal cortex, but not in the hippocampus. Conversely, chronic corticosterone administration increased *fndc5* expression in the frontal cortex, but not in the hippocampus. Social isolation in mice did not result in altered *fndc5* expression in either frontal cortex or hippocampus. Finally, fluoxetine, but not other antidepressants, increased *fndc5* gene expression in the mouse frontal cortex. Results indicate a region-specific modulation of *fndc5* in depressive-like behavior and by antidepressant in mice. Our finding of decreased prefrontal cortex *fndc5* expression in MDD individuals differs from results in mice, highlighting the importance of carefully interpreting observations in mice. The reduction in *fndc5* mRNA suggests that decreased central FNDC5/irisin could comprise a shared pathologic mechanism between MDD and AD.

## Significance Statement

Major depressive disorder (MDD) is a major cause of disability in humans. Physical exercise reduces the incidence and severity of MDD, but molecular mechanisms are elusive. One of the pleiotropic actions of exercise alludes to the increased production and circulation of irisin, a myokine cleaved from fibronectin type III domain-containing protein 5 (FNDC5) that mediates some benefits of exercise in the brain. Here, we observed reduced *fndc5* expression in postmortem samples of dorsolateral prefrontal cortex from patients with MDD. In the mouse frontal cortex, the modulation of *fndc5* was variable across models of depressive-like behavior. Our findings indicate reduced *fndc5* expression in MDD with discordant results in mice and stimulate further research on the roles of brain FNDC5/irisin in MDD.

## Introduction

Major depressive disorder (MDD) is a debilitating disease that affects one in five people during their lifetimes (Kessler et al., 2006). In addition to an immediate impact on daily activities, MDD contributes to the allostatic load, elevates the risk of developing other conditions, such as diabetes mellitus and dementia, and increases the odds of suicide by 20-fold (Otte et al., 2016; Selles et al., 2018; De Felice et al., 2022).

Irisin is a myokine that is increased in the bloodstream after physical exercise (Boström et al., 2012; Jedrychowski et al., 2015). Irisin derives from proteolytic processing of the transmembrane precursor fibronectin type III domain-containing protein 5 (FNDC5), a protein notably expressed in skeletal muscle and in select brain regions, including the hippocampus and frontal cortex (Boström et al., 2012; Wrann et al., 2013; Lourenco et al., 2019; Islam et al., 2021). Physical exercise and muscle contraction also induce *fndc5* expression in the brain (Wrann et al., 2013; Maekawa et al., 2018).

FNDC5/irisin regulates peripheral energy metabolism (Boström et al., 2012) and induces neuronal brain-derived neurotrophic factor (BDNF) expression (Wrann et al., 2013; Lourenco et al., 2022). In the brain, FNDC5/irisin was shown to be important for synapse plasticity and memory (Lourenco et al., 2019) and to promote exercise-induced neurogenesis.

FNDC5/irisin is reduced in postmortem Alzheimer’s disease (AD) brains and in mouse models of AD (Lourenco et al., 2019), and reduced CSF irisin is associated with impaired cognition in mild cognitive impairment and AD patients. Moreover, irisin counteracted molecular pathologic changes responsible for synapse failure and memory loss, and rescues cognition in mouse models of AD (de Freitas et al., 2020; Islam et al., 2021; Lourenco et al., 2019, 2022).

Physical exercise has been long known for its health benefits. Regular physical exercise improves general metabolism and prevents cardiovascular and neurologic disorders (Balducci et al., 2009; Mattson, 2012). In the brain, physical exercise induces a plethora of signals that boost neuronal health, synaptic plasticity, and neurogenesis (Isaac et al., 2021), but the precise molecular mechanisms remain unresolved. Exercise has antidepressant effects in MDD patients, notably in mild to moderate cases (Schuch et al., 2016, 2018). Evidence from rodent models indicates that exercise increases brain levels of monoamines, BDNF, and neurogenesis—all factors presumably associated with antidepressant actions (Krishnan and Nestler, 2008; Isaac et al., 2021). Importantly, irisin mediates brain benefits of physical exercise in mice (Lourenco et al., 2019; Islam et al., 2021). However, evidence for how and which myokines contribute to antidepressant actions in the brain is still lacking.

Depression is a risk factor and a comorbidity of AD. Biological processes that are common in depression and AD include vascular disease, alterations in glucocorticoid and neurotrophin signaling, hippocampal atrophy, neuroinflammation, and deficits in neurogenesis (Selles et al., 2018; Dafsari and Jessen, 2020). However, specific molecular mechanisms underlying the association between depression and AD have not been completely elucidated. Further, associations between AD neuropathology and depression remain controversial (Li et al., 2017; Babulal et al., 2020; Saldanha et al., 2021; Pomara et al., 2022).The links between AD and depression (Ledo et al., 2016, 2013; Dafsari and Jessen, 2020) and previous findings of the association between exercise and antidepressant activity prompted us to investigate whether *fndc5* expression could be dysregulated in depression. Here, we used several mouse models of depressive-like behavior and postmortem human brain tissue to test the hypothesis that depressive states impair *fndc5* expression in brain areas relevant to depression.

## Materials and Methods

### Human samples

Postmortem human dorsolateral prefrontal cortex RNA samples were donated by the Stanley Medical Research Institute Brain Bank (USA). The cohort comprised 36 patients diagnosed with MDD, MDD with psychotic features (MDD-P), or healthy control subjects (HCs). Patients were mostly white (94.4%), males represented between 50% and 66.6% of each group, and the mean age varied between 41.5 and 46.8 years (without significant statistical differences among groups). The mean postmortem interval (PMI) was 23.6 h for MDD patients, 25.3 h for HCs, and 35.75 h for MDD-P patients (Table 1). Causes of death in the control group included cardiac conditions (7 of 11 patients) and vehicle accidents (2 of 11 patients). Causes of death in MDD and MDD-P groups were mostly suicide, but also included cardiac conditions (3 of 10 patients in the MDD group). More detailed demographic information regarding this cohort can be found at https://www.stanleyresearch.org/brain-research/depression-collection. Postmortem hippocampal samples from subjects were not available at the time of this study.

**Table 1:** Demographic information about human brain samples used in this study

	HC	MDD	MDD-P	Significance (test performed)
Sample size	11	10	12	
Sex (F/M)	4/7	5/5	6/6	*p *=* *0.0716 (χ^2^ test)
Age (years)	47.91 (12.06)	44.70 (10.11)	41.50 (12.02)	*F*_(2,30)_ = 0.8925; *p *=* *0.4202 (one-way ANOVA × HC)
Race (% white)	90.91	100.00	91.67	*p *=* *0.0105 (χ^2^ test)
Duration of Illness (years)	—	11.11 (8.61)	12.33 (7.00)	*p *=* *0.7211 (χ^2^ test)
Brain pH	6.64 (0.19)	6.69 (0.14)	6.59 (0.14)	*F*_(2,30)_ = 1.075; *p *=* *0.3540 (one-way ANOVA × HC)
PMI (h)	24.73 (11.00)	23.40 (7.37)^0.949^	35.75 (13.96)^0.049^	*F*_(2,30)_ = 4.085; *p *=* *0.0270 (one-way ANOVA × HC)
Suicide (%)	0.00	60.00	75.00	*p *<* *0.0001 (χ^2^ test)
Antidepressant use (%)	9.09	90.00	58.33	*p *<* *0.0001 (χ^2^ test)
Antipychotic use (%)	0.00	30.00	41.66	*p *<* *0.0001 (χ^2^ test)

F, Female; M, male. Values represent the mean (SD). For PMI, one-way ANOVA with Dunnett’s post-test was performed, and respective adjusted *p* values are shown in superscript in MDD and MDD-P.

### RNA extraction and quantitative RT-PCR

For human samples, total RNA was extracted postmortem from dorsolateral prefrontal cortex (dlPFC; Brodmann’s area 46) from the Depression collection was provided by the Stanley Medical Research Institute Brain Bank (http://www.stanleyresearch.org/brain/). RNA was extracted from frozen brain tissue homogenized in TRIzol (Thermo Fisher Scientific) at the Stanley Medical Research Institute, following manufacturer instructions. Real-time quantitative RT-PCR (qRT-PCR) was performed with a high-capacity cDNA reverse transcription kit (Thermo Fisher Scientific) using 2.5 μg of RNA from each sample. For animal tissue, total RNA was extracted from frozen brain tissue homogenized in TRIzol (Thermo Fisher Scientific) with an ultrasonic homogenizer (Thermo Fisher Scientific), following manufacturer instructions. RNA purity and integrity were assessed by the 260:280 absorbance ratio, and only preparations with ratios ≥1.8 were used. For qRT-PCR, 1 μg of RNA was used for cDNA synthesis using a high-capacity cDNA reverse transcription kit (Thermo Fisher Scientific). Quantitative expression analysis of target genes was performed on a 7500 Real-Time PCR System (Thermo Fisher Scientific) with the PowerUp SYBR Green PCR Master Mix (Thermo Fisher Scientific).

For mouse samples, β-actin [*actb*; forward (Fw): TGTGACGTTGACATCCGTAAA; reverse (Rv): GTACTTGCGCTCAGGAGGAG] was used as an endogenous reference gene. For human samples, the geometric mean of β-actin (*actb*; Fw: GCACCCAGCACAATGAAG; Rv: CTTGCTGATCCACAT) and GAPDH (*gapdh*; Fw: CCTGTTCGACAGTCAGCCG; Rv: CGACCAAATCCGTTGACTCC) were used as endogenous references. Specific mouse *fndc5* (Fw: GGACTCTTGGAAAACACCACTG; Rv: TCCACACAGATGATCTCACCAC) and human *fndc5* (Fw: AAGCACAAGGACTGACTCAAGC; Rv: CATGTCCTTGATGGCTGGAT) primers were used. qRT-PCR was performed in 20 μl reactions, according to manufacturer instructions. Cycle threshold (C_t_) values were used to calculate the fold change in expression relative to control using the 2^-ΔΔCt^ method (Livak and Schmittgen, 2001).

### Animals

Male C57BL/6 mice were obtained from the animal facility at Federal University of Rio de Janeiro. Mice were 3–5 months of age at the beginning of experiments. Animals were housed in groups of up to five per cage, with free access to water and food, under a 12 h light/dark cycle (7:00 A.M. to 7:00 P.M. local time), and controlled room temperature and humidity. Behavioral tests were performed between zeitgeber time 4 (ZT4) and ZT8, equivalent to local time 11:00 A.M. to 5:00 P.M. All procedures followed principles of animal care and were previously approved by the Federal University of Rio de Janeiro Committee for Ethics and Animal Use, under protocol no. 020/18.

Trained experimentalists killed the mice by cervical dislocation between ZT6 and ZT11. Decapitation and tissue collection were performed immediately after cervical dislocation. Although all mice were killed during the light cycle, possible circadian variations of *fndc5* levels were not controlled for in this study.

We acknowledge that the use of male mice only is a limitation of our study. The use of female mice has been overlooked historically in preclinical neuroscience research, likely contributing to less effective diagnosis and treatment for women (Shansky, 2019; Shansky and Murphy, 2021). Investigating sex as a biological variable in the patterns of *fndc5* expression in depression in future studies is essential to understand its function and translational potential of our findings.

### Induction of depressive-like behavior in mice

#### Lipopolysaccharide administration

Male C57BL/6 mice (age, 3–4 months) received a single intraperitoneal injection of *Escherichia coli* lipopolysaccharide (LPS; 1 mg/kg) or an equivalent volume of saline solution as vehicle (Walker et al., 2013). The tail suspension test (TST) was performed 24 h after injection. Mice were killed ∼6 h after the tail suspension test, and tissue was collected and stored frozen at −80°C until molecular analysis. Sucrose preference testing was initiated immediately after the intraperitoneal injection. In this case, tissue was collected ∼50 h after a single LPS injection.

#### Chronic corticosterone administration

Male C57BL/6 mice (age, 3–5 months) were given a solution of 50 μg/ml corticosterone in 1% ethanol in drinking water available *ad libitum* for a period of 21 d. The control group received 1% ethanol in drinking water. Water bottles from both groups were weighted regularly to assess intake, and there were no significant differences in mean volumes consumed between groups. TST was performed on day 22, followed by a sucrose preference test on days 23–24. Mice were killed on day 25, and tissue was collected and stored at −80°C for molecular analyses.

#### Social isolation

Male C57BL/6 mice (age, 3 months) were placed in home cages in groups of five (control group) or alone (social isolation) for 60 d. Food and water were available *ad libitum* for both groups, and cages were kept in the animal facility with controlled light/dark cycles, temperature, and humidity. A tail suspension test was performed on day 61, and mice were killed and tissue was collected and stored the following day.

### Behavioral testing

#### Tail suspension test

Mice were suspended by their tails at a height of 1 m, in an apparatus where they were unable to escape, touch any surfaces, or see other mice. They were kept in this position for 6 min, and immobility time was scored by a trained experimentalist, as a measure of helplessness. Immobility was defined by a lack of escape-related behavior (e.g., swinging). After testing, immobility time during the final 3 min of testing was compared between groups (Can et al., 2011).

#### Sucrose preference test

Mice were individually placed in home cages with free access to food and with two identical bottles of water. On day 1, both bottles contained water (for habituation), and consumption of liquid from each bottle was measured, with no differences observed for any side or total volume between groups. On the following day, the content of one of the bottles was replaced by a solution of 2% sucrose in water. After 12 h, the volume of liquid remaining in each bottle was measured, and bottle positions were switched to avoid any preference for the location. The remaining volume was measured again after another period of 12 h, and total intake was calculated to determine the ratio of preference for the sucrose solution. Reduced sucrose preference is indicative of anhedonia, a marker of depressive-like behavior (Nasca et al., 2013).

### Antidepressant treatment

Male C57BL/6 mice (age, 3–4 months) received daily intraperitoneal injections of fluoxetine (10 mg/kg; Sigma-Aldrich) for 10 d. Clomipramine (10 mg/kg; Sigma-Aldrich), trazodone (40 mg/kg; Sigma-Aldrich), or an equivalent volume of saline was administered for 14 d. For ketamine, a single intraperitoneal injection (3 mg/kg; Syntec) was performed 3 h before killing. All drugs were diluted in sterile saline solution before injection.

### Statistical analysis

All analyses were performed on GraphPad Prism 7 or 8 (GraphPad Software). Datasets were assessed for group variance before statistical testing. Outliers were identified (using the ROUT method, set at 1%) before statistical testing, and outliers were excluded from the analysis. Values are expressed as the mean ± SEM unless stated otherwise in figure legends. Two-tailed statistical tests were used, and *p*-values are displayed above the bars. Post-test corrections were used every time multiple comparisons were performed, as indicated in the figure legends.

## Results

### Expression of fndc5 is reduced in postmortem brain tissue from MDD patients

**Figure 1.**
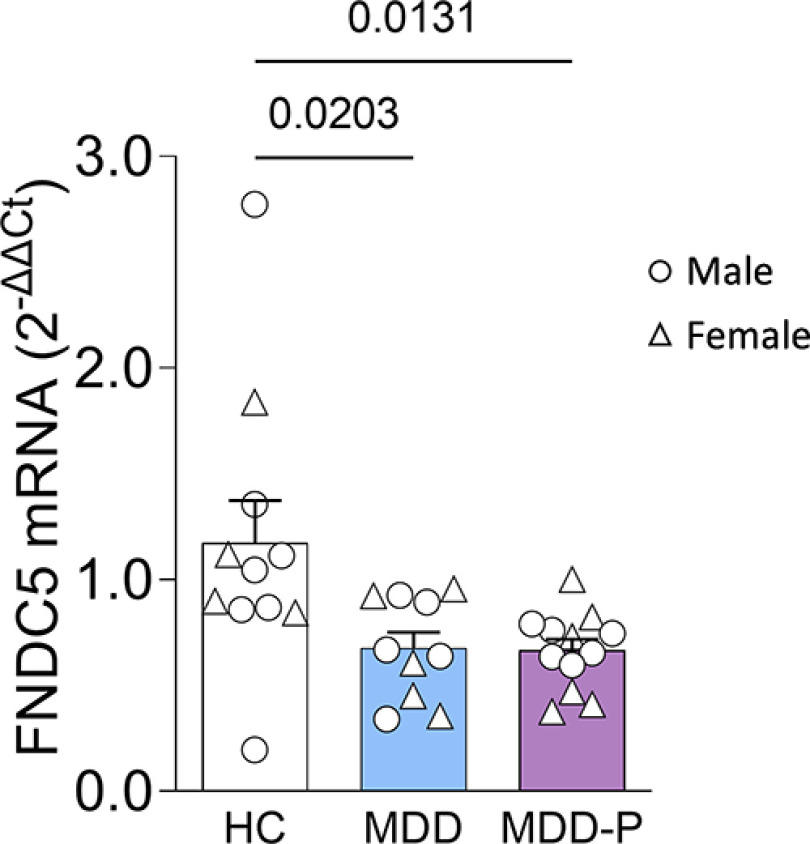
Expression of *fndc5* is reduced in the dorsolateral PFC of individuals with MDD. Expression of *fndc5* was measured in postmortem dorsolateral prefrontal cortex samples from individuals diagnosed with MDD (*N* = 10) or MDD-P (*N* = 12), or from HCs (*N* = 11). Reduced expression of *fndc5* was verified in MDD or MDD-P individuals. Male subjects are represented by circles, and female subjects by triangles. One-way ANOVA (*F*_(2,30)_ = 5.278; *p* = 0.0109) with Dunnett’s correction; adjusted *p*-values are shown above the bars. Bars express the mean ± SEM. Sex or antidepressant use did not impact *fndc5* expression (Extended Data Fig. 1-1).

We first assessed *fndc5* expression in postmortem brain tissue from a well established MDD cohort from the Stanley Medical Research Institute Brain Bank, which included individuals diagnosed with MDD and MDD-P, and healthy control subjects. The mean patient age was between 41 and 47 years, and males and females were equally distributed among groups (Table 1, demographics). qRT-PCR analysis revealed a marked decrease in *fndc5* mRNA in both MDD and MDD-P patient groups compared with control subjects (Fig. 1). Sex did not affect *fndc5* expression in the PFC (Extended Data Fig. 1-1*A*). Although only one control subject took antidepressants and most of MDD and MDD-P patients were under antidepressant prescription, antidepressant medication did not impact PFC *fndc5* mRNA within diagnostic categories (Extended Data Fig. 1-1*B*). We note, however, that additional studies in larger cohorts split by antidepressant pharmacological class are required to determine the potential impact of antidepressants on PFC *fndc5* expression. To our knowledge, this is the first report to examine *fndc5* expression in the brains of patients diagnosed with major depression. This result prompted us to investigate *fndc5* expression in mouse models of depressive-like behavior.

10.1523/ENEURO.0256-22.2023.f1-1 Figure 1-1 Expression of *fndc5* is reduced in the dorsolateral PFC of individuals with MDD, regardless of sex or antidepressant use. ***A***, Expression of *fndc5* in dlPFC of male (M) and female (F) individuals diagnosed with MDD (*N* = 10; 5 M/5 F), MDD with psychotic features (MDD-P; *N* = 12; 6 M/6 F), or HCs (*N* = 11; 7 M/4 F). Two-way ANOVA revealed a significant effect of diagnosis (*F*_(2,27)_ = 4,526; *p* = 0,0202), but no effect of sex (*F*_(1,27)_ = 0.04083; *p* = 0.8414) or interaction between these variables (*F*_(2,27)_ = 0.01420; *p* = 0.9859). ***B***, Expression of *fndc5* in dlPFC of individuals who were taking [Yes (Y)] or not taking [Not (N)] antidepressants. Patients were diagnosed with MDD (*N* = 10; 1 N/9 Y) or MDD-P (*N* = 12; 5N/7Y), or HCs (*N* = 11; 10 N/1 Y). Yellow dots in the MDD-P group indicate patients taking antipsychotic medication, but no antidepressants. Two-way ANOVA showed no effect of medication use (*F*_(1,29)_ = 0.007216; *p* = 0.9329). Download Figure 1-1, TIF file.

### Frontal cortex fndc5 expression is decreased by LPS in male mice

**Figure 2.**
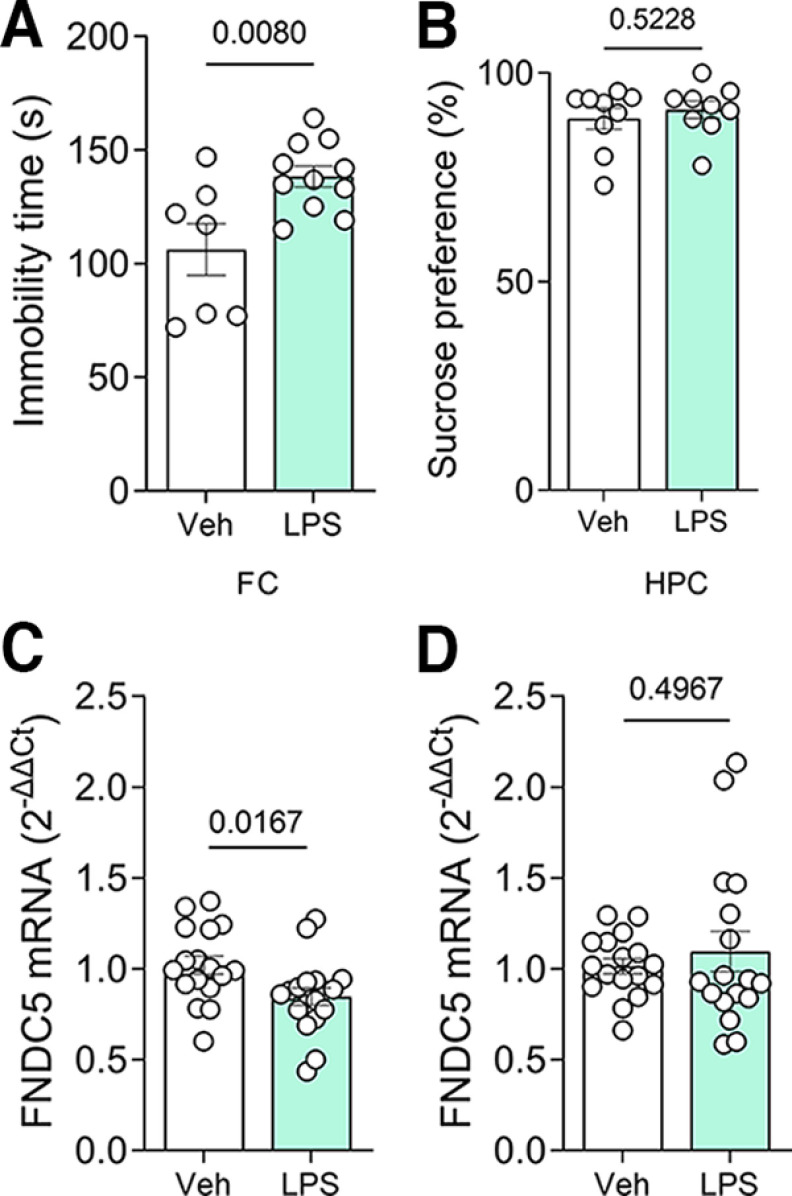
Lipopolysaccharide-induced depressive-like behavior decreased *fndc5* expression in the frontal cortex, but not the hippocampus. Male C57BL/6 mice received a single intraperitoneal injection of LPS (1 mg/kg), and their immobility times were assessed in the tail suspension test after 24 h. ***A***, Increased immobility times were observed in LPS-injected mice. ***B***, No difference was observed in sucrose preference in the sucrose consumption test. ***C***, ***D***, Analysis of *fndc5* mRNA levels revealed a decrease in the frontal cortex (***C***) and no changes in the hippocampus (***D***) of mice that received LPS. *N* = 7–18/group; Student’s *t* test. The *p*-values are depicted above the bars. Bars express the mean ± SEM.

We next investigated whether the induction of depressive-like behavior alters brain *fndc5* expression in male mice. Acute intraperitoneal administration of LPS (at 1 mg/kg) represents a classic model for the induction of depressive-like behavior in mice (O’Connor et al., 2009). We confirmed the depressive-like status 24 h after LPS administration by a marked increase in immobility in the TST (Fig. 2*A*), although mice did not develop anhedonic behavior in the sucrose preference test (Fig. 2*B*). Analysis of *fndc5* mRNA levels revealed decreased expression in the frontal cortex of LPS-treated mice (Fig. 2*C*), while the hippocampal expression remained unchanged (Fig. 2*D*).

### Fndc5 expression is increased in the mouse frontal cortex after chronic corticosterone administration

**Figure 3.**
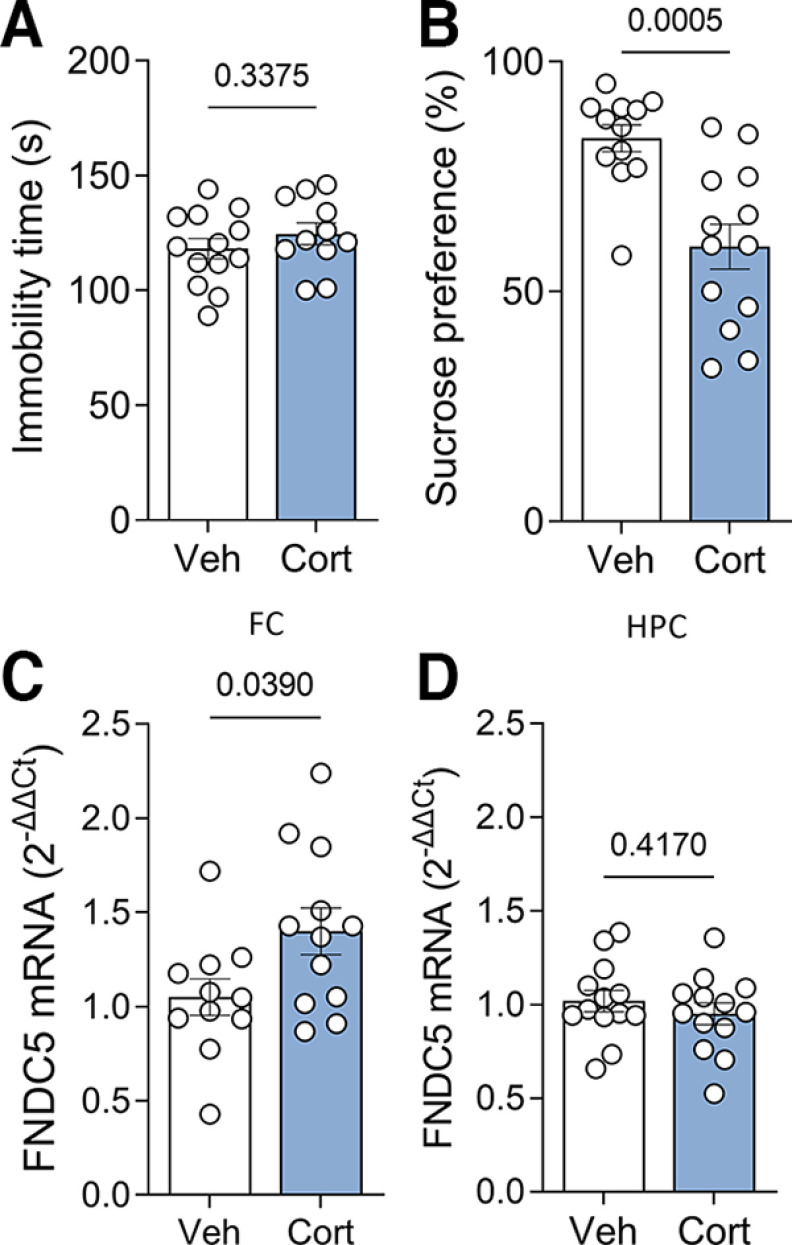
Chronic corticosterone-induced depressive-like behavior increase *fndc5* expression in the frontal cortex of mice. Male C57BL/6 mice were given corticosterone (50 μg/ml) or vehicle in the drinking water for 21 d. ***A***, No difference in the immobility time was observed in the tail suspension test. ***B***, Mice that received corticosterone failed to show sucrose preference in the sucrose consumption test. ***C***, ***D***, Analysis of *fndc5* mRNA levels showed increased levels in the frontal cortex (***C***) and no change in the hippocampus (***D***) of corticosterone-treated mice. Bars express the mean ± SEM. *N* = 11–13/group; Student’s *t* test. The *p*-value is shown above the bars. Bars express the mean ± SEM.

We further used chronic (21 d) administration of corticosterone in the drinking water (at 50 μg/ml) as an alternative protocol to chemically induce depressive-like behavior in mice, as previously described (Gourley et al., 2008). Immobility in the TST was similar between groups (Fig. 3*A*). On the other hand, male mice that received corticosterone showed no preference for sucrose in the sucrose consumption test (Fig. 3*B*), a proxy for the anhedonia component of depressive states (Nasca et al., 2015), as expected for this model (Ali et al., 2015). Corticosterone-treated male mice showed increased *fndc5* expression in the frontal cortex (Fig. 3*C*), while hippocampal *fndc5* expression was unchanged (Fig. 3*D*).

### Expression of fndc5 remains unchanged in social isolation-induced depressive-like behavior

**Figure 4.**
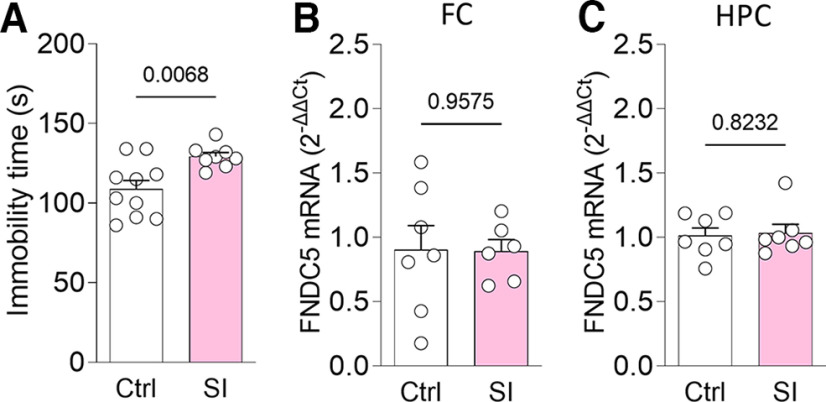
Social isolation-induced depressive-like behavior does not alter *fndc5* expression. Male C57BL/6 mice were housed in groups of five [controls (Ctrl)] or alone [social isolation (SI)] in their home cages for 60 d. ***A***, increased immobility times were observed in socially isolated mice compared with controls. ***B***, ***C***, Analysis of *fndc5* mRNA levels revealed no changes in frontal cortex (***B***) or hippocampus (***C***) between groups. *N* = 6–10; Student’s *t* test. The *p*-value is shown above the bars. Bars express the mean ± SEM.

To determine the impact of social stress on *fndc5* expression, we resorted to chronic social isolation, a protocol known to induce depressive-like behavior (Wallace et al., 2009). Male mice were either isolated or remained in groups for 60 d. Depressive-like behavior was confirmed by increased immobility in the TST (Fig. 4*A*). However, such mice showed no changes in *fndc5* expression in either frontal cortex (Fig. 4*B*) or hippocampus (Fig. 4*C*). The discrepant results observed in mice subjected to social isolation or treated with LPS or corticosterone suggest that distinct murine models of depressive-like behavior may not fully recapitulate the changes in *fndc5* expression observed in humans with MDD.

### Chronic fluoxetine administration selectively induces fndc5 expression in the frontal cortex of mice

**Figure 5.**
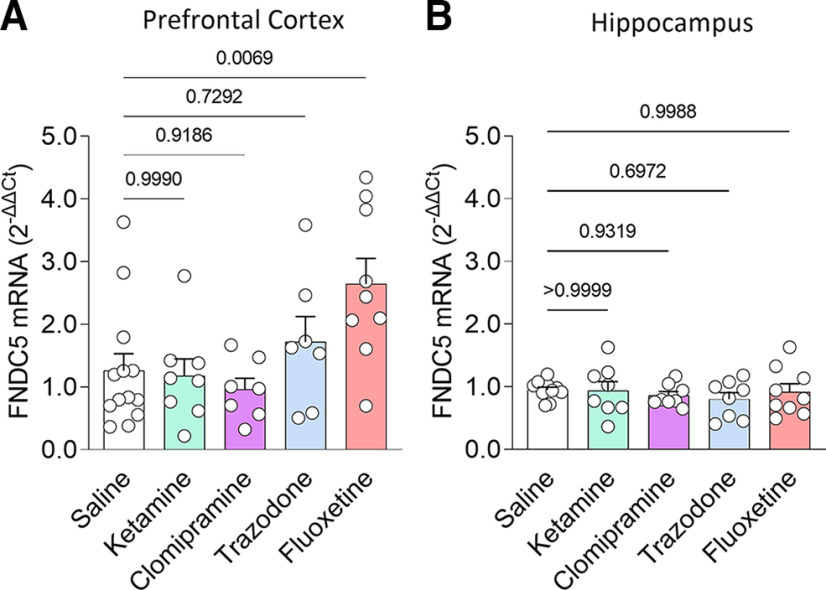
Treatment with fluoxetine selectively induces FNDC5 expression in the frontal cortex, but not in the hippocampus. ***A***, ***B***, Levels of *fndc5* mRNA in the frontal cortex (***A***) and hippocampus (***B***) of male C57BL/6 mice chronically treated with different antidepressants. For frontal cortex: saline (*N* = 13), ketamine (*N* = 8), clomipramine (*N* = 7), trazodone (*N* = 7), and fluoxetine (*N* = 9). *F*_(4,39)_ = 4.301; *p* = 0.0056. For hippocampus: saline (*N* = 11), ketamine (*N* = 8), clomipramine (*N* = 8), trazodone (*N* = 8), and fluoxetine (*N* = 9). *F*_(4,39)_ = 0.4019; *p* = 0.8061; one-way ANOVA with Dunnett’s correction. The adjusted *p*-value is shown above bars. Bars express the mean ± SEM. The *y*-axis range was formatted to accommodate changes in *fndc5* values.

Finally, we investigated whether brain *fndc5* expression is modulated in response to antidepressant treatment. We treated male C57BL/6 mice with antidepressants with different mechanisms of action. Mice received daily intraperitoneal injections of clomipramine or trazodone (or saline) for 14 d, fluoxetine for 10 d, or a single dose of ketamine, all of which are regimens known to produce bioactive responses (Gideons et al., 2014; Duque et al., 2016; Halliday et al., 2017; Browne et al., 2018). Among the antidepressants tested, fluoxetine selectively induced an increase in *fndc5* mRNA levels in the frontal cortex (Fig. 5*A*). None of the antidepressants tested (ketamine, clomipramine, trazodone, or fluoxetine) promoted significant changes in *fndc5* expression in the hippocampus (Fig. 5*B*), a brain region also linked to depression (Tsankova et al., 2006). These results indicate that fluoxetine promotes *fndc5* gene expression selectively in the frontal cortex.

## Discussion

Here, we investigated whether brain *fndc5* expression is altered in individuals diagnosed with MDD and is modulated by depressive-like behavior or by antidepressant treatment in mice. *Fndc5* is expressed in the brain (Wrann et al., 2013; Lourenco et al., 2019) and has been reported to promote BDNF expression (Wrann et al., 2013), exercise-induced neurogenesis (Islam et al., 2021), synaptic plasticity, and memory (Lourenco et al., 2019; Islam et al., 2021). Furthermore, brain delivery of viral vectors driving the expression of *fndc5* confers neuroprotection in mouse models of AD (Lourenco et al., 2019).

In rodents, a chronic unpredictable stress protocol reduced levels of FNDC5/irisin in the hippocampus (Babaei et al., 2021; Wu et al., 2021). In addition, intracerebroventricular administration of irisin (0.5–1 ng) reduced immobility times in the tail suspension and forced swim tests in mice (Siteneski et al., 2018). While FNDC5 knock-out mice do not develop alterations in the elevated plus maze or TST when compared with wild-type littermates (Islam et al., 2021), whether brain region-specific *fndc5* expression impacts depressive-like behavior remains an intriguing question that warrants further investigation.

Previous studies have shown reduced plasma irisin in patients with depression after stroke (Tu et al., 2018) or coronary heart disease (Han et al., 2019). This association was not observed in a cohort of obese women with depression (Hofmann et al., 2016). Plasma irisin may undergo complex regulation by multiple tissues and metabolic states. Recent evidence suggests that peripheral irisin crosses the blood–brain barrier and reaches brain areas linked to cognition (Islam et al., 2021). Whether blood irisin levels are modified by depression in the absence of comorbidities in humans and the intricacies of plasma to brain irisin transport remain unknown. To avoid these potential sources of confusion and focus on the specific modulation of brain *fndc5*, we assessed gene expression as a proxy of local FNDC5/irisin induction.

We first measured *fndc5* expression in dlPFC samples of MDD patients from a well characterized cohort composed of adult subjects (Martins-De-Souza et al., 2012; Sabunciyan et al., 2012). We observed a significant reduction in *fndc5* mRNA content in the dlPFC of individuals with depression, regardless of the presence or absence of psychosis, compared with healthy control subjects. To the best of our knowledge, this is the first evidence of altered (reduced) brain *fndc5* expression in MDD patients. Of note, age is a particularly relevant parameter as CNS irisin levels are altered by age (Lourenco et al., 2019, 2020), and this was carefully controlled in the cohort used in the current study. Although PMI was significantly higher for MDD-P, this difference does not seem to impact *fndc5* expression, as we observed similar levels in MDD and MDD-P groups—and both are similarly reduced when compared with HCs. It is noteworthy that most cases of major depression in this cohort had suicide as the cause of death, which might indicate a more severe pathology and/or unresponsiveness to treatment. We feel that these results encourage future studies in larger cohorts, as well as translational research to ascertain the roles of FNDC5/irisin in the PFC.

We used three different mouse models of depressive-like behavior to explore how *fndc5* expression is modulated by depressive-like behavior. LPS and corticosterone are thought to induce depressive-like states through different mechanisms and lead to different behavioral outcomes (Gourley et al., 2008; O’Connor et al., 2009). We observed divergent changes in frontal cortex *fndc5* expression in mice treated with LPS or corticosterone, with no changes in the hippocampus. While LPS-treated mice showed a reduction in FC *fndc5* expression, similar to what we observed in humans, corticosterone treatment led to increased frontal cortex *fndc5* expression. Notably, LPS-based and corticosterone-based models also resulted in divergent behavioral outcomes, with LPS recapitulating the despair component of depressive-like behavior and corticosterone inducing anhedonia behavior. Intriguingly, inducing a depressive-like state by deprivation of social interaction had no impact on *fndc5* expression in either region. It is noteworthy that previous reports have indicated that chronic unpredictable stress protocols reduce hippocampal *fndc5* expression in mice (Babaei et al., 2021; Wu et al., 2021).

MDD is a complex pathology with heterogeneous manifestations in humans (Fried et al., 2022). Distinct animal models may better recapitulate biochemical and physiological aspects more specific to different risk factors, such as inflammation, hypothalamus–pituitary–adrenal axis deregulation or persistent environmental stress (Krishnan and Nestler, 2008; Otte et al., 2016). Thus, the contrasting results observed by us in animal models and postmortem samples may be a consequence of the limitations in modeling mood disorders in rodents, as extensively noted (Cryan and Holmes, 2005; Gururajan et al., 2019; Fried et al., 2022).

The prefrontal cortex is notably impaired in MDD patients and rodent models of depressive-like behavior (Pizzagalli and Roberts, 2022). In patients, aberrant spontaneous brain activity (Gong et al., 2020) and reduced cortical thickness (Schmaal et al., 2017) were observed in the PFC of MDD patients when compared with control subjects. In rodents, stress induces morphologic and functional changes in the prefrontal cortex (Cerqueira et al., 2005; Hains et al., 2009; Yuen et al., 2012), as well increased glial reactivity (Banasr and Duman, 2008; Banasr et al., 2010). Notably, exercise training improves PFC function (Voss et al., 2013). Thus, exploring the roles of FNDC5 in PFC function associated with mood control is an exciting question for further studies.

To investigate whether antidepressants modulate *fndc5* expression in male mice, we analyzed both frontal cortex and hippocampus, two brain regions related to depression (Krishnan and Nestler, 2008), and examined the effects of drugs from different antidepressant classes. We administered chronic treatments with a selective serotonin reuptake inhibitor (fluoxetine), a serotonin antagonist and reuptake inhibitor (trazodone), and a tricyclic antidepressant (clomipramine), as well as acute treatment with fast-acting ketamine. This experimental plan allowed us to pinpoint the following two levels of specificity: (1) a region-specific effect in the frontal cortex of male mice; and (2) antidepressant class specificity for fluoxetine among the drugs investigated. Using naive mice further allowed us to isolate the basal effects of antidepressants in the absence of potentially unclear responses induced by depressive stimuli. Results revealed that modulation of *fndc5* expression is not a shared mechanism among antidepressants but is robustly and selectively induced by fluoxetine in the frontal cortex. Except for ketamine, most antidepressants require chronic administration to produce antidepressant effects. We note that the doses and durations of treatment we used for each compound have been previously reported to be compatible with their neuromodulatory activities in mice (Gideons et al., 2014; Duque et al., 2016; Halliday et al., 2017; Browne et al., 2018). We further note that most of the MDD and MDD-P subjects studied here took antidepressants and/or antipsychotics, whereas only one control case reported use of an antidepressant. No clear difference in *fndc5* mRNA levels was observed between those receiving antidepressants and not receiving them, although the reduced sample size precludes statistical analyses at this point. Further studies are necessary to determine whether the modulation of *fndc5* expression could be related to differential responsiveness to antidepressants in patients.

Our findings support the notion that the induction of depressive-like behavior or antidepressant responses can modulate differential *fndc5* expression in the frontal cortex of male mice. Divergent results observed in the brains of MDD individuals and mouse models may be related to limitations of modeling mood disorders in rodents. Although our results in MDD patients did not reveal an impact of sex in the *fndc5* mRNA in the PFC, a limitation of our mouse study is the use of male mice only. Sex differences have been historically underappreciated in neuroscience (Shansky, 2019; Shansky and Murphy, 2021), and additional studies investigating sex as a biological variable in the patterns of *fndc5* expression and roles in depression are needed. Finally, future studies are warranted to determine the relevance of brain *fndc5* expression in humans, and whether and how this relates to the pathophysiology of major depression.

## Synthesis

Reviewing Editor: Leandro Vendruscolo, National Institute on Drug Abuse Decisions are customarily a result of the Reviewing Editor and the peer reviewers coming together and discussing their recommendations until a consensus is reached. When revisions are invited, a fact-based synthesis statement explaining their decision and outlining what is needed to prepare a revision will be listed below. The following reviewer(s) agreed to reveal their identity: Kay Nisbett.

Both reviewers expressed concerns that need to be addressed by the authors.

## References

[B1] Ali SH, Madhana RM, A KV, Kasala ER, Bodduluru LN, Pitta S, Mahareddy JR, Lahkar M (2015) Resveratrol ameliorates depressive-like behavior in repeated corticosterone-induced depression in mice. Steroids 101:37–42. 10.1016/j.steroids.2015.05.010 26048446

[B2] Babaei A, Nourshahi M, Fani M, Entezari Z, Jameie SB, Haghparast A (2021) The effectiveness of continuous and interval exercise preconditioning against chronic unpredictable stress: involvement of hippocampal PGC-1α/FNDC5/BDNF pathway. J Psychiatr Res 136:173–183. 10.1016/j.jpsychires.2021.02.006 33607579

[B3] Babulal GM, Roe CM, Stout SH, Rajasekar G, Wisch JK, Benzinger TLS, Morris JC, Ances BM (2020) Depression is associated with tau and not amyloid positron emission tomography in cognitively normal adults. J Alzheimers Dis 74:1045–1055. 10.3233/JAD-191078 32144985 PMC7183906

[B4] Balducci S, Zanuso S, Fernando F, Fallucca S, Fallucca F, Pugliese G (2009) Physical activity/exercise training in type 2 diabetes. The role of the Italian Diabetes and Exercise Study. Diabetes Metab Res Rev 25:S29–S33. 10.1002/dmrr.985 19662617

[B5] Banasr M, Duman RS (2008) Glial loss in the prefrontal cortex is sufficient to induce depressive-like behaviors. Biol Psychiatry 64:863–870. 10.1016/j.biopsych.2008.06.008 18639237 PMC2709733

[B6] Banasr M, Chowdhury GMI, Terwilliger R, Newton SS, Duman RS, Behar KL, Sanacora G (2010) Glial pathology in an animal model of depression: reversal of stress-induced cellular, metabolic and behavioral deficits by the glutamate-modulating drug riluzole. Mol Psychiatry 15:501–511. 10.1038/mp.2008.106 18825147 PMC3347761

[B7] Boström P, Wu J, Jedrychowski MP, Korde A, Ye L, Lo JC, Rasbach KA, Boström EA, Choi JH, Long JZ, Kajimura S, Zingaretti MC, Vind BF, Tu H, Cinti S, Højlund K, Gygi SP, Spiegelman BM (2012) A PGC1-α-dependent myokine that drives brown-fat-like development of white fat and thermogenesis. Nature 481:463–468. 10.1038/nature10777 22237023 PMC3522098

[B8] Browne CA, Falcon E, Robinson SA, Berton O, Lucki I (2018) Reversal of stress-induced social interaction deficits by buprenorphine. Int J Neuropsychopharmacol 21:164–174. 10.1093/ijnp/pyx079 29020387 PMC5793841

[B9] Can A, Dao DT, Terrillion CE, Piantadosi SC, Bhat S, Gould TD (2011) The tail suspension test. J Vis Exp (59):e3769. 10.3791/3769 PMC3353516 22315011

[B10] Cerqueira JJ, Pêgo JM, Taipa R, Bessa JM, Almeida OFX, Sousa N (2005) Morphological correlates of corticosteroid-induced changes in prefrontal cortex-dependent behaviors. J Neurosci 25:7792–7800. 10.1523/JNEUROSCI.1598-05.2005 16120780 PMC6725252

[B11] Cryan JF, Holmes A (2005) The ascent of mouse: advances in modelling human depression and anxiety. Nat Rev Drug Discov 4:775–790. 10.1038/nrd1825 16138108

[B12] Dafsari FS, Jessen F (2020) Depression—an underrecognized target for prevention of dementia in Alzheimer’s disease. Transl Psychiatry 10:160. 10.1038/s41398-020-0839-1 32433512 PMC7239844

[B13] De Felice FG, Gonçalves RA, Ferreira ST (2022) Impaired insulin signalling and allostatic load in Alzheimer disease. Nat Rev Neurosci 23:215–230. 10.1038/s41583-022-00558-9 35228741

[B14] de Freitas GB, Lourenco MV, De Felice FG (2020) Protective actions of exercise-related FNDC5/Irisin in memory and Alzheimer’s disease. J Neurochem 155:602–611. 10.1111/jnc.15039 32396989

[B15] Duque A, Vinader-Caerols C, Monleón S (2016) Effects of social stress and clomipramine on emotional memory in mice. Acta Neurobiol Exp (Wars) 76:225–233. 10.21307/ane-2017-022 27685775

[B16] Fried EI, Flake JK, Robinaugh DJ (2022) Revisiting the theoretical and methodological foundations of depression measurement. Nat Rev Psychol 1:358–368. 10.1038/s44159-022-00050-2 PMC10723193 38107751

[B17] Gideons ES, Kavalali ET, Monteggia LM (2014) Mechanisms underlying differential effectiveness of memantine and ketamine in rapid antidepressant responses. Proc Natl Acad Sci U S A 111:8649–8654. 10.1073/pnas.1323920111 24912158 PMC4060670

[B18] Gong J, Wang J, Qiu S, Chen P, Luo Z, Wang J, Huang L, Wang Y (2020) Common and distinct patterns of intrinsic brain activity alterations in major depression and bipolar disorder: voxel-based meta-analysis. Transl Psychiatry 10:353. 10.1038/s41398-020-01036-5 33077728 PMC7573621

[B19] Gourley SL, Kiraly DD, Howell JL, Olausson P, Taylor JR (2008) Acute hippocampal brain-derived neurotrophic factor restores motivational and forced swim performance after corticosterone. Biol Psychiatry 64:884–890. 10.1016/j.biopsych.2008.06.016 18675955 PMC2633780

[B20] Gururajan A, Reif A, Cryan JF, Slattery DA (2019) The future of rodent models in depression research. Nat Rev Neurosci 20:686–701. 10.1038/s41583-019-0221-6 31578460

[B21] Hains AB, Vu MAT, Maciejewski PK, van Dyck CH, Gottron M, Arnsten AFT (2009) Inhibition of protein kinase C signaling protects prefrontal cortex dendritic spines and cognition from the effects of chronic stress. Proc Natl Acad Sci U S A 106:17957–17962. 10.1073/pnas.0908563106 19805148 PMC2742406

[B22] Halliday M, Radford H, Zents KAM, Molloy C, Moreno JA, Verity NC, Smith E, Ortori CA, Barrett DA, Bushell M, Mallucci GR (2017) Repurposed drugs targeting eIF2α-P-mediated translational repression prevent neurodegeneration in mice. Brain 140:1768–1783. 10.1093/brain/awx074 28430857 PMC5445255

[B23] Han W, Zhang C, Wang H, Yang M, Guo Y, Li G, Zhang H, Wang C, Chen D, Geng C, Jiang P (2019) Alterations of irisin, adropin, preptin and BDNF concentrations in coronary heart disease patients comorbid with depression. Ann Transl Med 7:298–298. 10.21037/atm.2019.05.77 31475168 PMC6694242

[B24] Hofmann T, Elbelt U, Ahnis A, Obbarius A, Rose M, Klapp BF, Stengel A (2016) The exercise-induced myokine irisin does not show an association with depressiveness, anxiety and perceived stress in obese women. J Physiol Pharmacol 67:195–203. 27226179

[B25] Isaac AR, Lima-Filho R, Lourenco MV (2021) How does the skeletal muscle communicate with the brain in health and disease? Neuropharmacology 197:108744. 10.1016/j.neuropharm.2021.108744 34363812

[B26] Islam MR, et al. (2021) Exercise hormone irisin is a critical regulator of cognitive function. Nat Metab 3:1058–1070. 10.1038/s42255-021-00438-z 34417591 PMC10317538

[B27] Jedrychowski MPP, Wrann CDD, Paulo JAA, Gerber KKK, Szpyt J, Robinson MMM, Nair KSS, Gygi SPP, Spiegelman BMM (2015) Detection and quantitation of circulating human irisin by tandem mass spectrometry. Cell Metab 22:734–740. 10.1016/j.cmet.2015.08.001 26278051 PMC4802359

[B28] Kessler RC, Akiskal HS, Ames M, Birnbaum H, Greenberg P, Hirschfeld RMA, Jin R, Merikangas KR, Simon GE, Wang PS (2006) Prevalence and effects of mood disorders on work performance in a nationally representative sample of U.S. workers. Am J Psychiatry 163:1561–1568. 10.1176/ajp.2006.163.9.1561 16946181 PMC1924724

[B29] Krishnan V, Nestler EJ (2008) The molecular neurobiology of depression. Nature 455:894–902. 10.1038/nature07455 18923511 PMC2721780

[B30] Ledo JH, Azevedo EP, Clarke JR, Ribeiro FC, Figueiredo CP, Foguel D, De Felice FG, Ferreira ST (2013) Amyloid-β oligomers link depressive-like behavior and cognitive deficits in mice. Mol Psychiatry 18:1053–1054. 10.1038/mp.2012.168 23183490 PMC3781315

[B31] Ledo JH, Azevedo EP, Beckman D, Ribeiro FC, Santos LE, Razolli DS, Kincheski GC, Melo HM, Bellio M, Teixeira AL, Velloso LA, Foguel D, De Felice FG, Ferreira ST (2016) Cross talk between brain innate immunity and serotonin signaling underlies depressive-like behavior induced by alzheimer’s amyloid-oligomers in mice. J Neurosci 36:12106–12116. 10.1523/JNEUROSCI.1269-16.2016 27903721 PMC6601978

[B32] Li P, Hsiao I-T, Liu C-Y, Chen C-H, Huang S-Y, Yen T-C, Wu K-Y, Lin K-J (2017) Beta-amyloid deposition in patients with major depressive disorder with differing levels of treatment resistance: a pilot study. EJNMMI Res 7:24. 10.1186/s13550-017-0273-4 28324341 PMC5360749

[B33] Livak KJ, Schmittgen TD (2001) Analysis of relative gene expression data using real-time quantitative PCR and the 2−ΔΔCT method. Methods 25:402–408. 10.1006/meth.2001.1262 11846609

[B34] Lourenco MV, et al. (2019) Exercise-linked FNDC5/irisin rescues synaptic plasticity and memory defects in Alzheimer’s models. Nat Med 25:165–175. 10.1038/s41591-018-0275-4 30617325 PMC6327967

[B35] Lourenco MV, Ribeiro FC, Sudo FK, Drummond C, Assunção N, Vanderborght B, Tovar‐Moll F, Mattos P, De Felice FG, Ferreira ST (2020) Cerebrospinal fluid irisin correlates with amyloid‐β, BDNF, and cognition in Alzheimer’s disease. Alzheimers Dement (Amst) 12:e12034. 10.1002/dad2.12034 32582833 PMC7306518

[B36] Lourenco MV, de Freitas GB, Raony Í, Ferreira ST, De Felice FG (2022) Irisin stimulates protective signaling pathways in rat hippocampal neurons. Front Cell Neurosci 16:953991. 10.3389/fncel.2022.953991 36187295 PMC9518673

[B37] Maekawa T, Ogasawara R, Tsutaki A, Lee K, Nakada S, Nakazato K, Ishii N (2018) Electrically evoked local muscle contractions cause an increase in hippocampal BDNF. Appl Physiol Nutr Metab 43:491–496. 10.1139/apnm-2017-0536 29558209

[B38] Martins-De-Souza D, Guest PC, Harris LW, Vanattou-Saifoudine N, Webster MJ, Rahmoune H, Bahn S (2012) Identification of proteomic signatures associated with depression and psychotic depression in post-mortem brains from major depression patients. Transl Psychiatry 2:e87. 10.1038/tp.2012.13 22832852 PMC3309534

[B39] Mattson MP (2012) Energy intake and exercise as determinants of brain health and vulnerability to injury and disease. Cell Metab 16:706–722. 10.1016/j.cmet.2012.08.012 23168220 PMC3518570

[B40] Nasca C, Xenos D, Barone Y, Caruso A, Scaccianoce S, Matrisciano F, Battaglia G, Mathe AA, Pittaluga A, Lionetto L, Simmaco M, Nicoletti F (2013) L-acetylcarnitine causes rapid antidepressant effects through the epigenetic induction of mGlu2 receptors. Proc Natl Acad Sci U S A 110:4804–4809. 10.1073/pnas.1216100110 23382250 PMC3607061

[B41] Nasca C, Bigio B, Zelli D, Nicoletti F, McEwen BS (2015) Mind the gap: glucocorticoids modulate hippocampal glutamate tone underlying individual differences in stress susceptibility. Mol Psychiatry 20:755–763. 10.1038/mp.2014.96 25178162 PMC4366364

[B42] O’Connor JC, Lawson MA, André C, Moreau M, Lestage J, Castanon N, Kelley KW, Dantzer R (2009) Lipopolysaccharide-induced depressive-like behavior is mediated by indoleamine 2,3-dioxygenase activation in mice. Mol Psychiatry 14:511–522. 10.1038/sj.mp.4002148 18195714 PMC2683474

[B43] Otte C, Gold SM, Penninx BW, Pariante CM, Etkin A, Fava M, Mohr DC, Schatzberg AF (2016) Major depressive disorder. Nat Rev Dis Primers 2:16065. 10.1038/nrdp.2016.65 27629598

[B44] Pizzagalli DA, Roberts AC (2022) Prefrontal cortex and depression. Neuropsychopharmacology 47:225–246. 10.1038/s41386-021-01101-7 34341498 PMC8617037

[B45] Pomara N, Bruno D, Plaska CR, Ramos-Cejudo J, Osorio RS, Pillai A, Imbimbo BP, Zetterberg H, Blennow K (2022) Plasma amyloid-β dynamics in late-life major depression: a longitudinal study. Transl Psychiatry 12:301. 10.1038/s41398-022-02077-8 35902554 PMC9334636

[B46] Sabunciyan S, Aryee MJ, Irizarry RA, Rongione M, Webster MJ, Kaufman WE, Murakami P, Lessard A, Yolken RH, Feinberg AP, Potash JB, Consortium GRED (2012) Genome-wide DNA methylation scan in major depressive disorder. PLoS One 7:e34451. 10.1371/journal.pone.0034451 22511943 PMC3325245

[B47] Saldanha NM, Suemoto CK, Rodriguez RD, Leite REP, Nascimento C, Ferreti-Rebustini R, da Silva MM, Pasqualucci CA, Nitrini R, Jacob-Filho W, Lafer B, Grinberg LT, Nunes PV (2021) β-amyloid pathology is not associated with depression in a large community sample autopsy study. J Affect Disord 278:372–381. 10.1016/j.jad.2020.09.062 33007627

[B48] Schmaal L, et al. (2017) Cortical abnormalities in adults and adolescents with major depression based on brain scans from 20 cohorts worldwide in the ENIGMA Major Depressive Disorder Working Group. Mol Psychiatry 22:900–909. 10.1038/mp.2016.60 27137745 PMC5444023

[B49] Schuch FB, Vancampfort D, Richards J, Rosenbaum S, Ward PB, Stubbs B (2016) Exercise as a treatment for depression: a meta-analysis adjusting for publication bias. J Psychiatr Res 77:42–51. 10.1016/j.jpsychires.2016.02.023 26978184

[B50] Schuch FB, Vancampfort D, Firth J, Rosenbaum S, Ward PB, Silva ES, Hallgren M, D Leon AP, Dunn AL, Deslandes AC, Fleck MP, Carvalho AF, Stubbs B (2018) Physical activity and incident depression: a meta-analysis of prospective cohort studies. Am J Psychiatry 175:631–648. 10.1176/appi.ajp.2018.17111194 29690792

[B51] Selles MC, Oliveira MM, Ferreira ST (2018) Brain inflammation connects cognitive and non-cognitive symptoms in Alzheimer’s disease. J Alzheimers Dis 64:S313–S327. 10.3233/JAD-179925 29710716

[B52] Shansky RM (2019) Are hormones a “female problem” for animal research? Science 364:825–826. 10.1126/science.aaw7570 31147505

[B53] Shansky RM, Murphy AZ (2021) Considering sex as a biological variable will require a global shift in science culture. Nat Neurosci 24:457–464. 10.1038/s41593-021-00806-8 33649507 PMC12900283

[B54] Siteneski A, Cunha MP, Lieberknecht V, Pazini FL, Gruhn K, Brocardo PS, Rodrigues ALS (2018) Central irisin administration affords antidepressant-like effect and modulates neuroplasticity-related genes in the hippocampus and prefrontal cortex of mice. Prog Neuropsychopharmacol Biol Psychiatry 84:294–303. 10.1016/j.pnpbp.2018.03.004 29524513

[B55] Tsankova NM, Berton O, Renthal W, Kumar A, Neve RL, Nestler EJ (2006) Sustained hippocampal chromatin regulation in a mouse model of depression and antidepressant action. Nat Neurosci 9:519–525. 10.1038/nn1659 16501568

[B56] Tu W, Qiu H, Liu Q, Li X, Zhao J, Zeng X (2018) Decreased level of irisin, a skeletal muscle cell-derived myokine, is associated with post-stroke depression in the ischemic stroke population. J Neuroinflammation 15:133. 10.1186/s12974-018-1177-6 29720216 PMC5932807

[B57] Voss MW, Vivar C, Kramer AF, van Praag H (2013) Bridging animal and human models of exercise-induced brain plasticity. Trends Cogn Sci 17:525–544. 10.1016/j.tics.2013.08.001 24029446 PMC4565723

[B58] Walker AK, Budac DP, Bisulco S, Lee AW, Smith RA, Beenders B, Kelley KW, Dantzer R (2013) NMDA receptor blockade by ketamine abrogates lipopolysaccharide-induced depressive-like behavior in C57BL/6J mice. Neuropsychopharmacology 38:1609–1616. 10.1038/npp.2013.71 23511700 PMC3717543

[B59] Wallace DL, Han M-H, Graham DL, Green TA, Vialou V, Iñiguez SD, Cao J-L, Kirk A, Chakravarty S, Kumar A, Krishnan V, Neve RL, Cooper DC, Bolaños CA, Barrot M, McClung CA, Nestler EJ (2009) CREB regulation of nucleus accumbens excitability mediates social isolation–induced behavioral deficits. Nat Neurosci 12:200–209. 10.1038/nn.2257 19151710 PMC2721778

[B60] Wrann CD, White JP, Salogiannnis J, Laznik-Bogoslavski D, Wu J, Ma D, Lin JD, Greenberg ME, Spiegelman BM (2013) Exercise induces hippocampal BDNF through a PGC-1α/FNDC5 pathway. Cell Metab 18:649–659. 10.1016/j.cmet.2013.09.008 24120943 PMC3980968

[B61] Wu Y, Sun F, Guo Y, Zhang Y, Li L, Dang R, Jiang P (2021) Curcumin relieves chronic unpredictable mild stress-induced depression-like behavior through the PGC-1α/FNDC5/BDNF pathway. Behav Neurol 2021:2630445. 10.1155/2021/2630445 34950248 PMC8692045

[B62] Yuen EY, Wei J, Liu W, Zhong P, Li X, Yan Z (2012) Repeated stress causes cognitive impairment by suppressing glutamate receptor expression and function in prefrontal cortex. Neuron 73:962–977. 10.1016/j.neuron.2011.12.033 22405206 PMC3302010

