# Supplementary figures and images for: Brain FNDC5/Irisin Expression in Patients and Mouse Models of Major Depression
Source: eNeuro. 2023 Feb 13;10(2):ENEURO.0256-22.2023. doi: 10.1523/ENEURO.0256-22.2023 (PMC9927507; doi:10.1523/ENEURO.0256-22.2023)

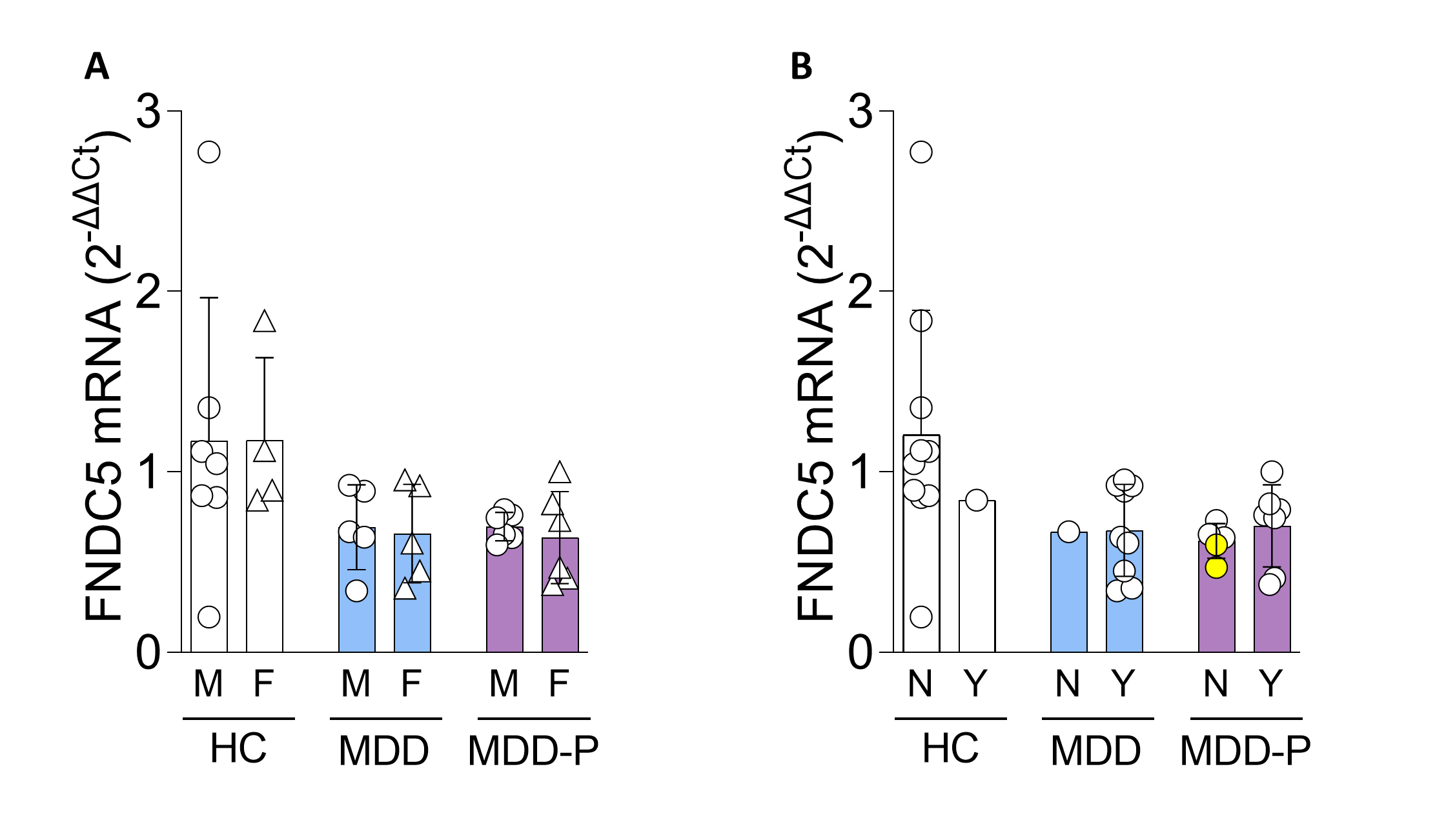

Supplement: Figure 1-1 — Expression of fndc5 is reduced in the dorsolateral PFC of individuals with MDD, regardless of sex or antidepressant use. A, Expression of fndc5 in dlPFC of male (M) and female (F) individuals diagnosed with MDD (N = 10; 5 M/5 F), MDD with psychotic features (MDD-P; N = 12; 6 M/6 F), or HCs (N = 11; 7 M/4 F). Two-way ANOVA revealed a significant effect of diagnosis (F(2,27) = 4,526; p = 0,0202), but no effect of sex (F(1,27) = 0.04083; p = 0.8414) or interaction between these variables (F(2,27) = 0.01420; p = 0.9859). B, Expression of fndc5 in dlPFC of individuals who were taking [Yes (Y)] or not taking [Not (N)] antidepressants. Patients were diagnosed with MDD (N = 10; 1 N/9 Y) or MDD-P (N = 12; 5N/7Y), or HCs (N = 11; 10 N/1 Y). Yellow dots in the MDD-P group indicate patients taking antipsychotic medication, but no antidepressants. Two-way ANOVA showed no effect of medication use (F(1,29) = 0.007216; p = 0.9329). Download Figure 1-1, TIF file. [file enu-eN-NWR-0256-22-s01.tif]
